# Variation in forest root image annotation by experts, novices, and AI

**DOI:** 10.1186/s13007-024-01279-z

**Published:** 2024-10-01

**Authors:** Grace Handy, Imogen Carter, A. Rob Mackenzie, Adriane Esquivel-Muelbert, Abraham George Smith, Daniela Yaffar, Joanne Childs, Marie Arnaud

**Affiliations:** 1 Birmingham Institute of Forest Research, University of Birmingham, Birmingham, UK; 2 School of Geography Earth and Environmental Sciences, University of Birmingham, Birmingham, UK; 3 Department of Computer Science, University of Copenhagen, Copenhagen, Denmark; 4 Environmental Science Division, Oak Ridge National Laboratory, Oak Ridge, TN USA; 5 Institute of Ecology and Environmental Sciences (IEES), CNRS, INRAE, Sorbonne Université, Paris, France

**Keywords:** Roots, Root annotation, Machine learning, Minirhizotron, Image analysis, Forests

## Abstract

**Background:**

The manual study of root dynamics using images requires huge investments of time and resources and is prone to previously poorly quantified annotator bias. Artificial intelligence (AI) image-processing tools have been successful in overcoming limitations of manual annotation in homogeneous soils, but their efficiency and accuracy is yet to be widely tested on less homogenous, non-agricultural soil profiles, e.g., that of forests, from which data on root dynamics are key to understanding the carbon cycle. Here, we quantify variance in root length measured by human annotators with varying experience levels. We evaluate the application of a convolutional neural network (CNN) model, trained on a software accessible to researchers without a machine learning background, on a heterogeneous minirhizotron image dataset taken in a multispecies, mature, deciduous temperate forest.

**Results:**

Less experienced annotators consistently identified more root length than experienced annotators. Root length annotation also varied between experienced annotators. The CNN root length results were neither precise nor accurate, taking ~ 10% of the time but significantly overestimating root length compared to expert manual annotation (*p* = 0.01). The CNN net root length change results were closer to manual (*p* = 0.08) but there remained substantial variation.

**Conclusions:**

Manual root length annotation is contingent on the individual annotator. The only accessible CNN model cannot yet produce root data of sufficient accuracy and precision for ecological applications when applied to a complex, heterogeneous forest image dataset. A continuing evaluation and development of accessible CNNs for natural ecosystems is required.

## Background

Artificial intelligence (AI) methods have transformed the scientific community in recent years due to their ability to automate the collection and preparation of empirical data which could previously only be carried out manually [1]. In ecology, studies most commonly adopt the use of convolutional neural networks (CNNs), a type of deep learning model well suited for analysing visual data, for identification of individuals or species from images, video, and sound [2]. Image segmentation, the identification of individual pixels as belonging to a particular class, is increasingly being applied successfully to understand complex ecological systems [3–6]. A continuing synergy between AI and ecology could greatly improve data throughput for the understanding and restoration of ecosystems in the face of global change [7].

Fine root systems are complex belowground systems which are an important driver of ecosystem responses to global change, as they are fundamental components of the carbon cycle and represent ~ 1/3 of net primary production (NPP) on a global scale [8]. The amount of carbon stored as root biomass [9] or released into the soil through autotrophic and heterotrophic respiration depends on root dynamics (i.e., ‘birth’, growth, death) and decomposition rates [10–12]. Studies investigating root dynamics are essential for the comprehensive understanding of global carbon budgets [13, 14] but remain rare due to the methodological difficulty in studying the dynamics of belowground plant tissue across ecosystems [15–17]. Non-destructive techniques that allow for root dynamics to be observed by photographing the same points in the soil profile to obtain data on fine root production, mortality, and turnover rates include rhizoboxes, root observation windows and minirhizotrons [18, 19]. Minirhizotrons are a widely used in-situ technique where images are taken periodically at fixed points along Perspex tubes fixed into the ground [18, 20–22]. A study by Jose et al. [23] indicated that minirhizotron and soil core techniques produce comparable results, displaying how minirhizotrons can be used to overcome some of the limitations of the labour intensive, destructive, and non-repeatable traditional techniques such as excavation and coring [19, 24].

Root image analysis on images collected from root observation techniques typically require detection and quantification of the pixels depicting root tissue, via an image-analysis process usually known as segmentation [25, 26]. Traditionally, this has relied on humans to identify roots and trace each length and diameter by hand using root tracing software tools such as Rootfly (Wells and Birchfield, Clemson University, SC, USA) [27]. However, the employment of manual annotation has drawbacks including the significant time investment required (1–1.5 h per 100 cm^2^ of image) and the potential for observer bias [28, 29]. It is not uncommon for long-term experiments to produce tens of thousands of images for analysis [18, 30, 31]. Inevitably, this could result in an analysis bottleneck, preventing the full potential of the root observation method being reached, because the images are taken orders of magnitude faster than manual root annotation can be carried out.

The potential for observer bias could be driven largely by variability in individual knowledge, training, and experience, and may result in inconsistencies between and within studies when root annotation is carried out by different individuals [32]. This may be particularly significant in long-term experiments such as Free Air Carbon Enrichment studies [33], which are likely to experience turnover of staff over the years, and often decades, of the experiment’s duration. However, observer variation has only been assessed in one very recent study based in fen peatland where novice annotators were found to be reporting 3x the root length of expert annotators [32]. The complexity of image analysis and magnitude of variation between individuals may be further increased with system complexity, including higher variation in soil moisture and structure, variation in soil colour (e.g., by soil horizon), more plant species, the presence of soil animals, and the presence of non-root plant litter [34]. Such system complexity may cause variation even between experienced image annotators. To overcome the limitations of manual annotation, recent technological developments have allowed for the automation of root annotation using CNNs, which have the potential to eliminate inter-observer bias and reduce the time required for image annotation [29, 35–37].

RootPainter, an accessible CNN software which has combined annotation, training, and segmentation with an easy-to-use interface [38], has been successfully used for automated segmentation of images from roots in simple crop systems [26, 31, 38–43] and to an extent in two natural ecosystems [34]. However, despite good correlations (*R*^*2*^ = 0.81 and 0.87) when comparing RootPainter and human annotation in Mediterranean tree-grass and temperate grassland, RootPainter still consistently overestimated root length [34]. Therefore, it remains unclear how accurately AI will perform when translated out of laboratory and agricultural sites and applied to more complex ecosystems, such as forests.

Quantifying the effect of inter-observer variation on manual annotation and understanding whether an accessible CNN can successfully be applied to a complex ecological system will help to improve the accuracy of future root dynamics studies. It will also promote research into the development and application of AI technology to complex ecosystems and aid their understanding and restoration in the face of global change. Here we used data from a long-term minirhizotron experiment in a multispecies, mature, deciduous forest with characteristically heterogeneous soil composition to (1) Quantify the variation in root annotation of different human annotators with various experience levels (2), Understand the variation in root annotation between the same human annotator before and after training and (3) Investigate potential differences between manual root annotation and AI (CNN model trained using RootPainter) at (i) a single time point (root length) and (ii) across a time series (root length net change).

## Methods

### Experimental setup: a mature forest with a complex belowground system

**Fig. 1:**
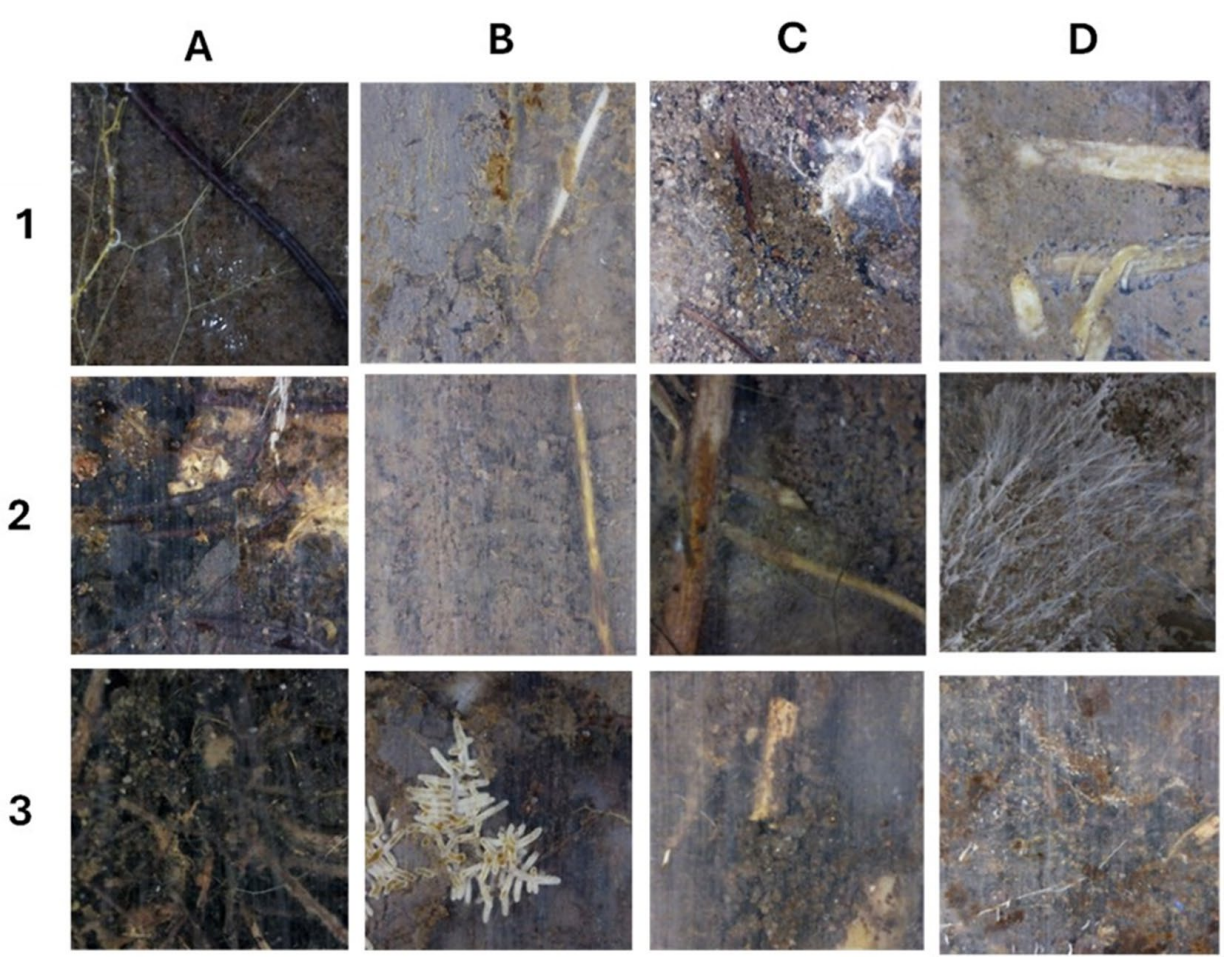
Example images from the minirhizotron image dataset, illustrating the variety and complexity of images obtained from the BIFoR FACE minirhizotrons

We used minirhizotron images taken under an old growth forest. These highly complex, ambiguous and heterogeneous images reflect the in-situ ‘natural’ variability of forest soils (Fig. 1). Images were collected at the Birmingham Institute of Forest Research Free Air Carbon Enrichment (BIFoR FACE) experimental site; a temperate, deciduous forest in central England (52.801°N, 2.301°W) with an Orthic Luvisol soil. This forest is composed of a Common hazel (*Corylus avellana* L.) dominated understory and a 180-year-old English oak (*Quercus robur* L.) upper canopy. Ground flora is sparse, composed primarily of bramble (*Rubus fruticosus* agg.), honeysuckle (*Lonicera periclymenum*), grasses (Poaceae spp.), ivy (*Hedera helix*), and bluebells (Hyacinthoides non-scripta (L.)), with naturally regenerating tree seedlings in canopy gaps. A full description of the experimental set up can be found at Hart et al. [33].

The images were taken from 14 minirhizotron tubes, 155 cm in length and 5.5 cm in diameter, installed at an angle of 40 ± 5°. Each minirhizotron tube was inserted perpendicular to an individual *Q. robur* tree, in areas free of any herbaceous species. The images are taken 2 cm apart, and a maximum of 70 images were taken per tube, equating to a maximum vertical depth of 80–100 cm. Images were taken in each tube once a month for 15-months, to build up a bank of 7935 minirhizotron images, from which the 3 image datasets used in this study were created (see image datasets subsection). The images were collected using a minirhizotron camera (MS-190-UHD Minirhizotron camera, Vienna Scientific Instruments, Vienna). Each image represents a 1.5 cm x 1.5 cm region of soil and was resized to 936 × 960 px so they were the correct size to be inputted into Rootfly for manual analysis.

Drivers of heterogeneity in the image dataset include variation in belowground biomass such as root species variation, with the potential for both tree and ground flora roots to be captured (Fig. 1) as well as the presence of ectomycorrhizal colonised root tips (B3, C1, Fig. 1), hyphae (A1, Fig. 1), and mycelium (D2, Fig. 1). Drivers in variation also include the presence of above-ground biomass, including decomposing matter i.e. twigs and leaves (A2, A3, C3, Fig. 1), and presence of soil fauna. Abiotic factors such as differing soil types, stones/pebbles, water pockets, and light/dark contrast also contribute to heterogeneity of the images in this dataset (Fig. 1). It is impossible to ensure perfect contact between minirhizotron tube and soil, not least because of networks of soil pores and channels. This can result in precipitation and, particularly in clay soil, streaking/smearing on the outside of the tube.

## Image datasets

Three datasets of images (one training dataset and two datasets of test images) were created from an existing 15-month bank of minirhizotron images from BIFoR FACE (Table 1).

**Table 1:** Description of the image datasets

Image dataset name	Number of images	Image selection	Dataset application
Training Dataset	966	Randomly selected from across all 14 tubes (1000 images originally selected, but images at the same window location as dataset 1 and 2 were removed).	Train the CNN model using RootPainter.
Dataset 1	30	Three images (one from bottom 30%, one from middle 40% and one from top 30%) were randomly selected from ten minirhizotron tubes.	Compare (i) manual root length annotation between different human annotators with different experience levels and (ii) manual and CNN annotation, at a single point in time.
Dataset 2	2116	Four months (Mar – Jun 2023) worth of images from 14 minirhizotron tubes (*n* = 529 images per month).	Compare manual and AI root length annotation across a time series.

## Manual annotators

To understand how root annotation varied between individuals with different experience levels and between individuals with the same experience level (first objective), we used several persons (hereafter called annotators), including students studying environmental science, technicians, and scientists. Level of annotator experience was determined mainly by previous experience annotating roots on images, but previous experience of working with intact roots (e.g. root washing, scanning, collecting) was also considered (Table 2).

**Table 2:** Description of the manual annotators

Experience category	Research experience	Number of annotators
Novice	< 50 root images annotated pre study	4
Novice_OE	< 50 root images annotated pre study but has > 500 h experience with intact roots	1
Experienced	> 1000 root images annotated pre study and > 8 years experience regularly annotating roots	3

## Methods for comparing manual annotation

To test for variation in manual annotation between and within experience levels, each annotator (*n* = 8) was provided dataset 1 (30 images). Here they were instructed to identify and trace what they considered to be live roots, which were absent of ectomycorrhizal colonisation, on each image. Each annotator used a common root annotation software (Rootfly, Wells and Birchfield, Clemson University, SC, USA) to annotate root length, diameter, and colour on minirhizotron images. To reduce variation because of unfamiliarity with the software, each annotator, regardless of previous experience, was provided with written, step-by-step instructions on how to use Rootfly. The deviation in root length output (mm) by Novice and Novice_OE from the Experienced annotators was then measured.

For our second objective, to understand how root annotation varied in the same individual before and after training, one originally Novice annotator spent 3 months after they first annotated dataset 1 working as a research technician manually tracing minirhizotron images using Rootfly. Their experience level therefore changed from Novice to Experienced. The annotator then repeated the root analysis process on dataset 1, and the average root length per image output (mm) of them as a Novice and an Experienced annotator was compared. This annotator then repeated the root analysis process a third time after 1 year as an experienced annotator to ascertain whether root annotation continued to change after further training or plateaued once experienced status was met.

## Methods for training the CNN

For our third objective, to investigate variations between manual and automated root annotation, a CNN model was trained in RootPainter using the training image data set. The CNN training process involved 15 h of corrective annotation, including both CNN training, image segmentation, and the human annotator viewing images and assigning corrections to the model segmentations. The CNN trained by RootPainter is a variant of U-Net [44] using Group norm [45] and implemented in PyTorch. The RootPainter training process trains a CNN via stochastic gradient descent with momentum. Internally RootPainter assigns approximately one out of every five annotated images to a validation set that is used to select the best model so far, which is used for subsequent segmentation tasks, including generating the segmentations that the user corrects in the interface and the final segmentations. A more detailed explanation of the RootPainter training process can be found in Smith et al. [38].

To speed up the training process, each image in the training dataset was cropped to a 50% sub region. Images are annotated with RootPainter using a human-in-the-loop process referred to as corrective annotation, where an annotator aims to correctively annotate all false positives and all false negatives in the model’s predicted segmentations for each image. Training was stopped once all images in the image training dataset had been annotated (*n* = 966). This was deemed an appropriate point to stop as the highest rolling dice score (*n* = 50) had not improved for the last ~ 200 images. A dice score measures the agreement between the image segmentation and the ground truth. In this context the ground truth is the corrected segmentation i.e. the segmentation with the users’ annotations of false positive and false negative regions applied.

## Methods for comparing manual annotation and CNN performance

The trained CNN model was applied to dataset 1 to extract the total root length per image. The deviation in root length output (mm) by the CNN model from the individual Experienced annotators was then measured.

To test the accuracy of the model output, we then compared total root length per image output from the CNN model with an expert consensus manual annotation. The expert consensus manual annotation is separate from the individual annotations. Every object that had been identified by at least one of the 8 individual annotators across test image set 1 was decided to be root or not root based on a majority agreement between the expert annotators. Expert consensus total root length was subsequently decided by this method. This expert consensus manual annotation was deemed to represent the consensus ground truth for the annotation of the images in test image set 1.

## Methods for comparing manual annotation and CNN performance over a time series

All images from test image set 2 were manually annotated in Rootfly by an experienced user, and root length per image was calculated by adding up the total lengths of the individual roots on each image. The CNN model was then applied to this same data set and the root length outputs per image from each month were compared to manual outputs. For each image, the net change in root length per image between month 1 and month 4 was calculated by subtracting total root length in month 1 from total root length in month 4, using both manual and CNN outputs. Net root length change across time was compared between the two techniques.

## Statistical analyses

All datasets were found to be not normally distributed after using the Shapiro-Wilk test. For research question one, a Lin’s concordance correlation coefficient was used to quantify the agreement, combining precision and accuracy, between annotators of the same experience level i.e. the agreement between the two most experienced annotators and agreement between the two least experienced annotators. For research question two to understand the variation in root annotation between the same human annotator before (as a Novice annotator) and after training (as an experienced annotator working 3 months as a part time research assistant and again after working 1 year as a part time research assistant), a Friedman test was used. Post hoc analysis with Wilcoxon signed-rank tests was conducted with a Bonferroni correction applied.

For research question three (i) to investigate potential differences between manual root annotation and AI (CNN model trained using RootPainter) at a single point in time (root length), a Wilcoxon signed-rank test was used to determine whether there was a significant difference between the root length output of the CNN model and the manually annotated expert consensus. A Lin’s concordance correlation coefficient was then used to quantify the agreement, combining precision and accuracy, between the manual and CNN root length outputs [46, 47].

For research question three, to compare the performance of an expert manual annotation and the CNN over a time series, a Wilcoxon signed-rank test was used to determine whether there was a significant difference between the total root length outputs of the CNN model and the manual annotation of an experienced Rootfly user in each of 4 consecutive months. A Lin’s concordance correlation coefficient was again used to quantify the agreement for precision and accuracy between the manual and CNN root length outputs. Finally, a Wilcoxon signed-rank test was used to determine whether there was a significant difference between the net change in root length outputs across a 4-month period of the CNN model and the manual annotation of an experienced Rootfly user.

## Results

### Results for comparing manual annotation and CNN performance

**Fig. 2:**
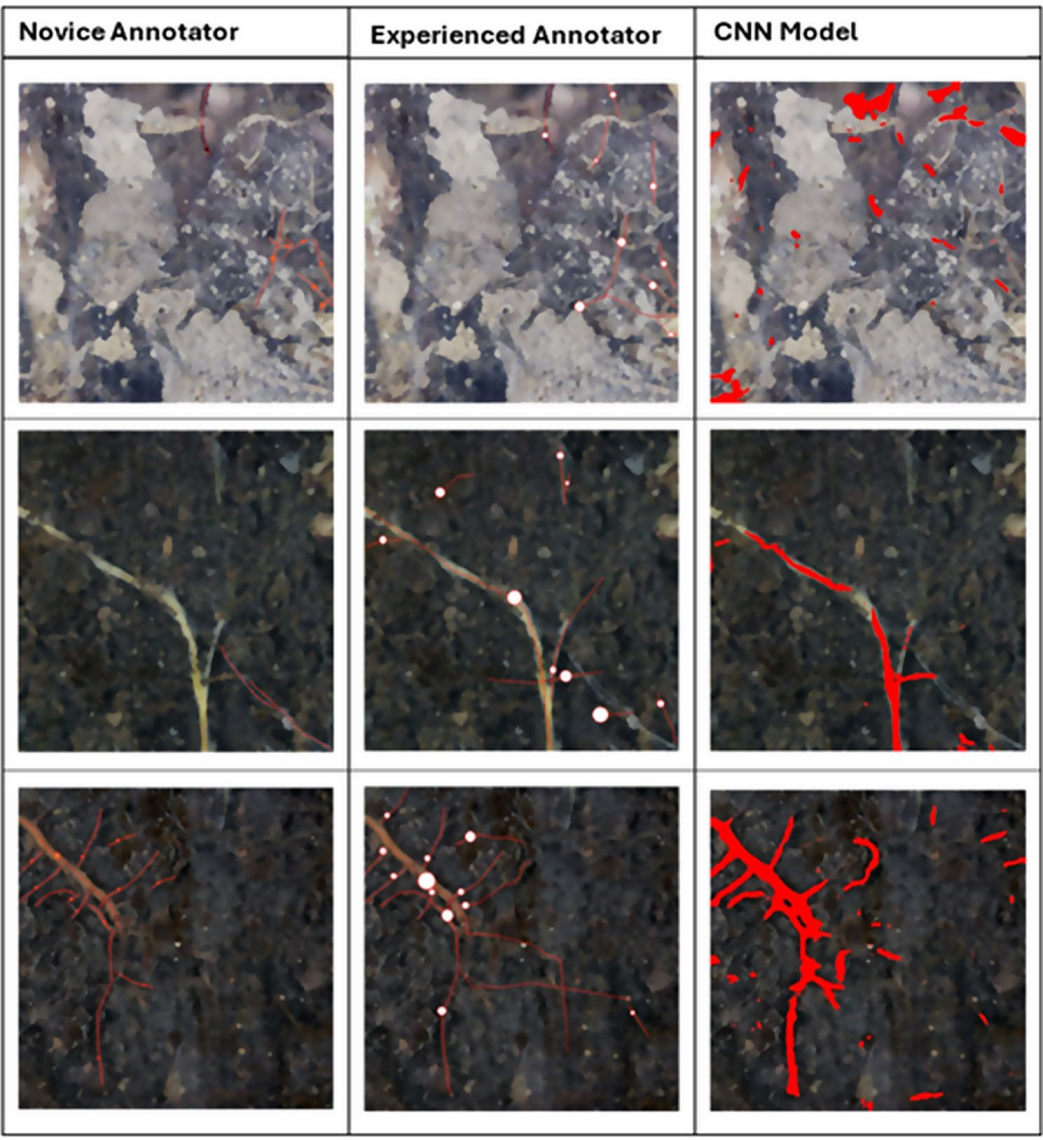
Examples of images annotated by a Novice Annotator, Experienced Annotator and CNN model. For the manual annotation, the red lines represent traced length, and the circles represent traced diameter. For the CNN model annotation, the red represents all areas where the model predicted root to be present. Row one; O Horizon, 2.6 cm vertical depth. Row two; A Horizon, 19 cm vertical depth. Row three; O Horizon, 2.7 cm vertical depth

The CNN model took ~ 160 person-hours (hours spent by a person training to use the software and trialling different ways to train the model on RootPainter) prior to training the final CNN model. It took ~ 15 CPU-hours (hours for which a computer’s central processing unit (CPU) was used for processing instructions of a computer program) to train and test the final model using 966 images.

**Fig. 3:**
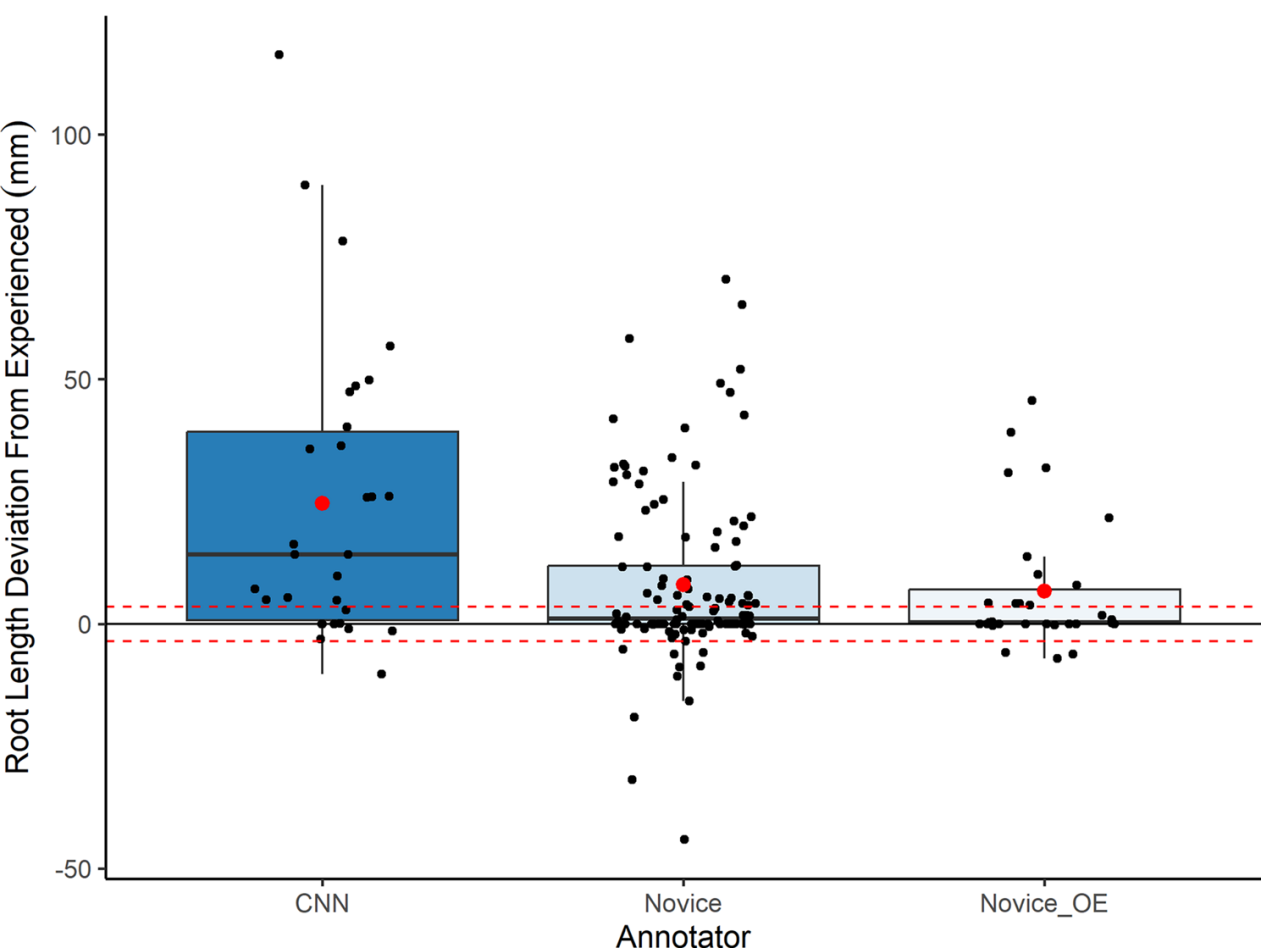
Deviation in root length annotation from experienced annotators by less experienced annotators and a CNN. Each point represents the difference in total root length estimated for an image (*n* = 30) by Novice (*n* = 3), Novice_OE (Image-analysis novice but with other root experience) (*n* = 1) and the CNN from the median of the root length estimated by the experienced annotators to the same image. The red dashed lines represent ± the median absolute variation (MAD) from the median estimation by experienced annotators

**Fig. 4:**
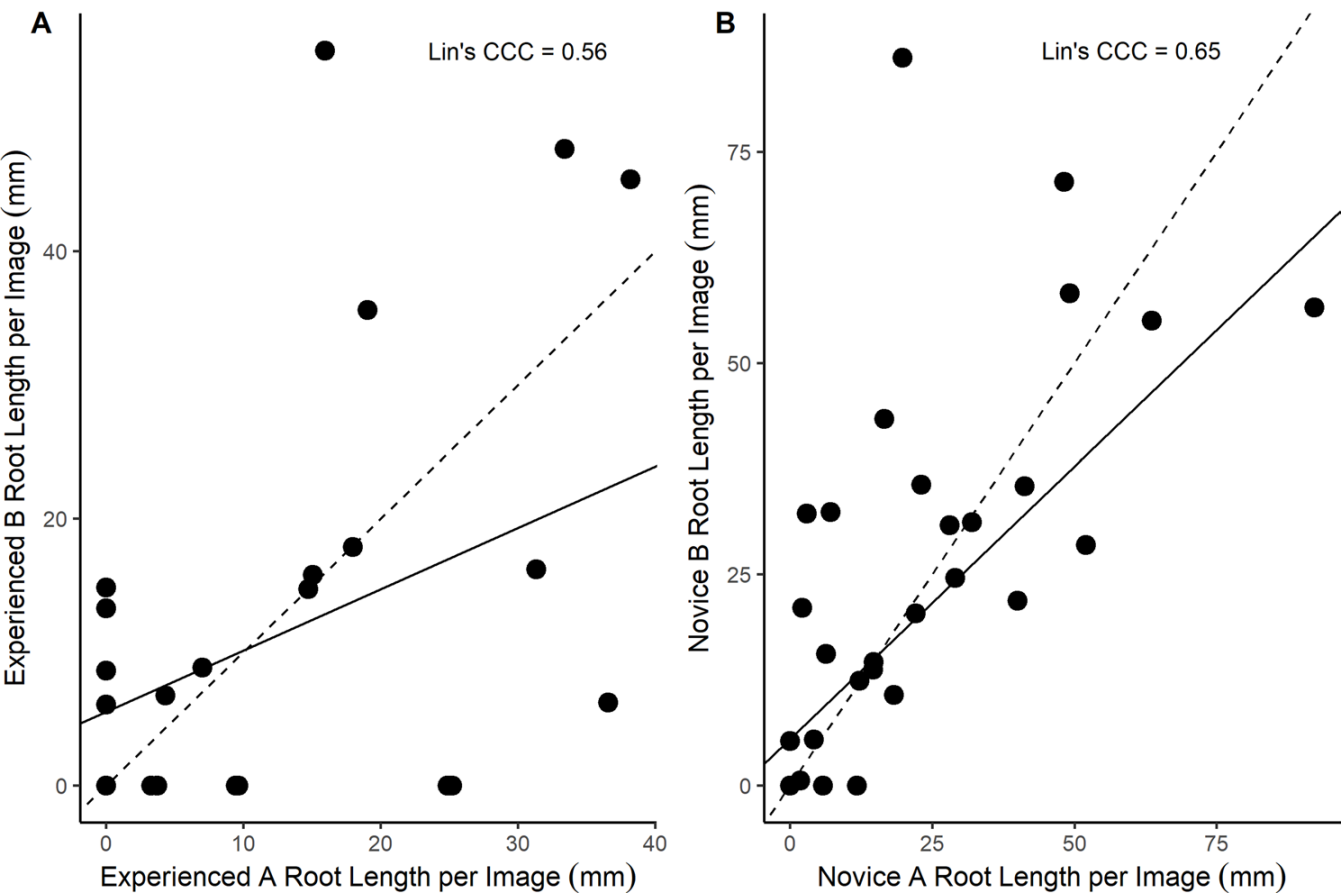
The relationship between root length annotation of the two most and two least experienced annotators. Annotation was carried out manually on the same images (*n* = 30) using Rootfly. Novice A; most novice, Novice B; second most novice; Experienced A; most experienced, Experienced B; second most experienced. Level of experience determined by the number of images previously annotated for roots. The dashed line is a 1:1 line and the solid line represents Lin’s CCC

The median deviation from the Experienced annotations by the Novice annotations (median increase of 1.1 mm (MAD ± 3.1)) was larger than the deviation by the Novice_OE (Image-analysis novice but with other root experience) annotations (Novice_OE median increase of 0.4 mm (MAD ± 2.4)), but neither were significant. However, the skewed distribution of the data indicates that deviations by the Novice and Novice_OE annotations from the Experienced annotations were mostly small but occasionally very large (Fig. 3). This was predominantly driven by over- rather than under-annotation (Fig. 3). The median deviation from the Experienced annotators by the CNN was the largest (median increase of 14.2 (MAD ± 14.2)) (Fig. 4), again driven by over-annotations. Segmentation by the CNN was also often unsatisfactory when inspected by eye (Fig. 2).

There was a statistically significant difference in root length annotation when image dataset 1 was annotated by the same individual, depending on their level of training, X^2^ [2] = 29.19, p = < 0.01. There was a significant reduction between root length output from when the individual was classified as a novice and after they had spent 3 months as a part time research assistant who worked as the principle annotator of minirhizotron images for this minirhizotron experiment (p = < 0.05), reducing from mean 21.9 mm (SE ± 4.1) and median 15.6 mm (MAD ± 13.1) to mean 8.4 mm (SE ± 2.2) and median 3.1 mm (MAD ± 3.1) of root annotated per image. There was also a significant reduction between when the individual was classified as a novice and after they had spent 1 year as a part time research assistant annotating roots on minirhizotron images (p = < 0.05), reducing to mean 10.6 mm (SE ± 2.9) and median 2.1 mm (MAD ± 2.1) per image. However, there was no statistically significant change between root length output between when the individual had spent 3 months as a part time research assistant annotating roots on minirhizotron images and when they had spent 1 year as a part time research assistant in the same role.

As well as variation between the experience groups, there was variation within the experience groups i.e. variation between the individual annotators within the experienced group and variation between the individual annotators within the Novice group. Novice annotators had an average range of root length output per image of 23.4 mm (SE ± 3.8) and the Experienced annotators had an average range of root length output per image of 9.2 mm (SE ± 2.1). When considering the two most and two least experienced annotators the concordance correlation coefficient quantifying the agreement for precision and accuracy was 0.65 between the two least experienced annotators (Fig. 4A) and 0.56 between the top two most experienced annotators (Fig. 4B). Depending on the descriptive scale used, these values of concordance can be described as poor to moderate [46, 47].

**Fig. 5:**
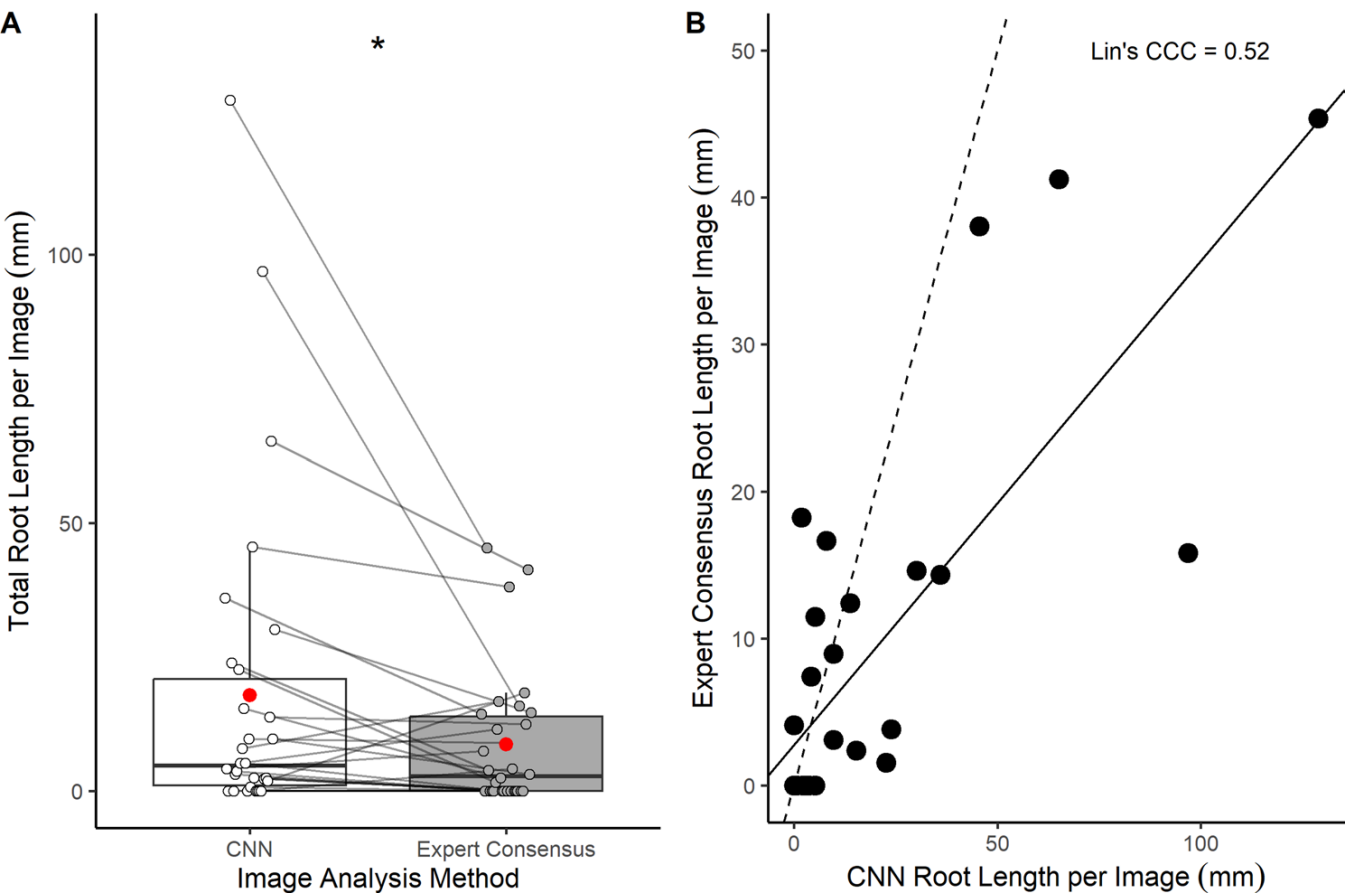
Root length comparison between an expert consensus manual annotation and CNN A Total root length output per image (*n* = 30) from an expert consensus manual annotation, and from a CNN model trained using RootPainter. Wilcoxon signed-rank test (v = 63, *p* = 0.01). **B** The relationship between total root length output per image (*n* = 30) from an expert consensus manual annotation and from a CNN model trained using RootPainter. The dashed line is a 1:1 line and the solid line represents Lin’s CCC

There was a significant difference between the total root length output per image from the expert consensus and the CNN (Fig. 5A). The CNN values for root length (mean = 17.9 mm and median = 4.7 mm) were almost double the human generated expert consensus (mean = 8.7 mm and median = 2.7 mm) (Fig. 5A). The concordance correlation coefficient quantifying the agreement for precision and accuracy between the manual and CNN root length outputs was 0.52 (Fig. 5B). These values of concordance can be described as poor to moderate [46, 47].

**Fig. 6:**
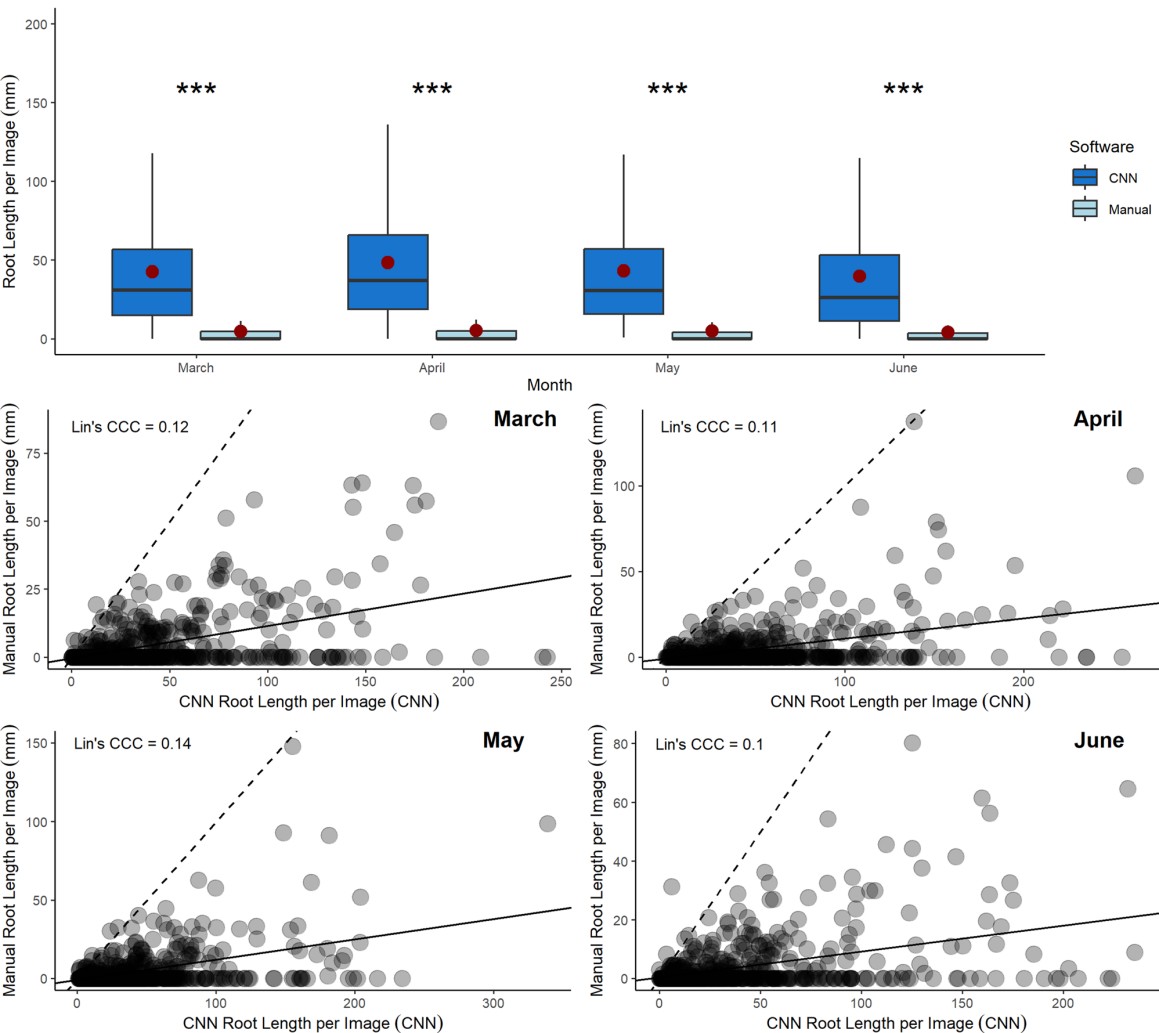
Root length comparison between an expert manual annotation and CNN in 4 consecutive months **A** Total root length per image (mm) (*n* = 529) from an expert consensus manual annotation and a CNN model trained using RootPainter. Annotated images were taken in the same locations in March, April, May and June. **B** The relationship between total root length output per image (*n* = 529) from an expert consensus manual annotation and from a CNN model trained using RootPainter, in March, April, May and June. The dashed lines are 1:1 lines and the solid lines represent Lin’s CCC

Measurements from CNN were consistently significantly higher (8–9 times) when compared to manually annotated measurements in every month of this study: March (V = 139522, p = < 0.0005), April (V = 139527, p = < 0.0005), May (V = 139423, p = < 0.0005) and June (V = 1392744, p = < 0.0005) (Fig. 6A). The concordance correlation coefficient quantifying the agreement between the manual and CNN root length outputs were 0.12 for March, 0.11 for April, 0.14 for May and 0.1 for June (Fig. 6B). These values of concordance can be described as poor [46, 47].

**Fig. 7:**
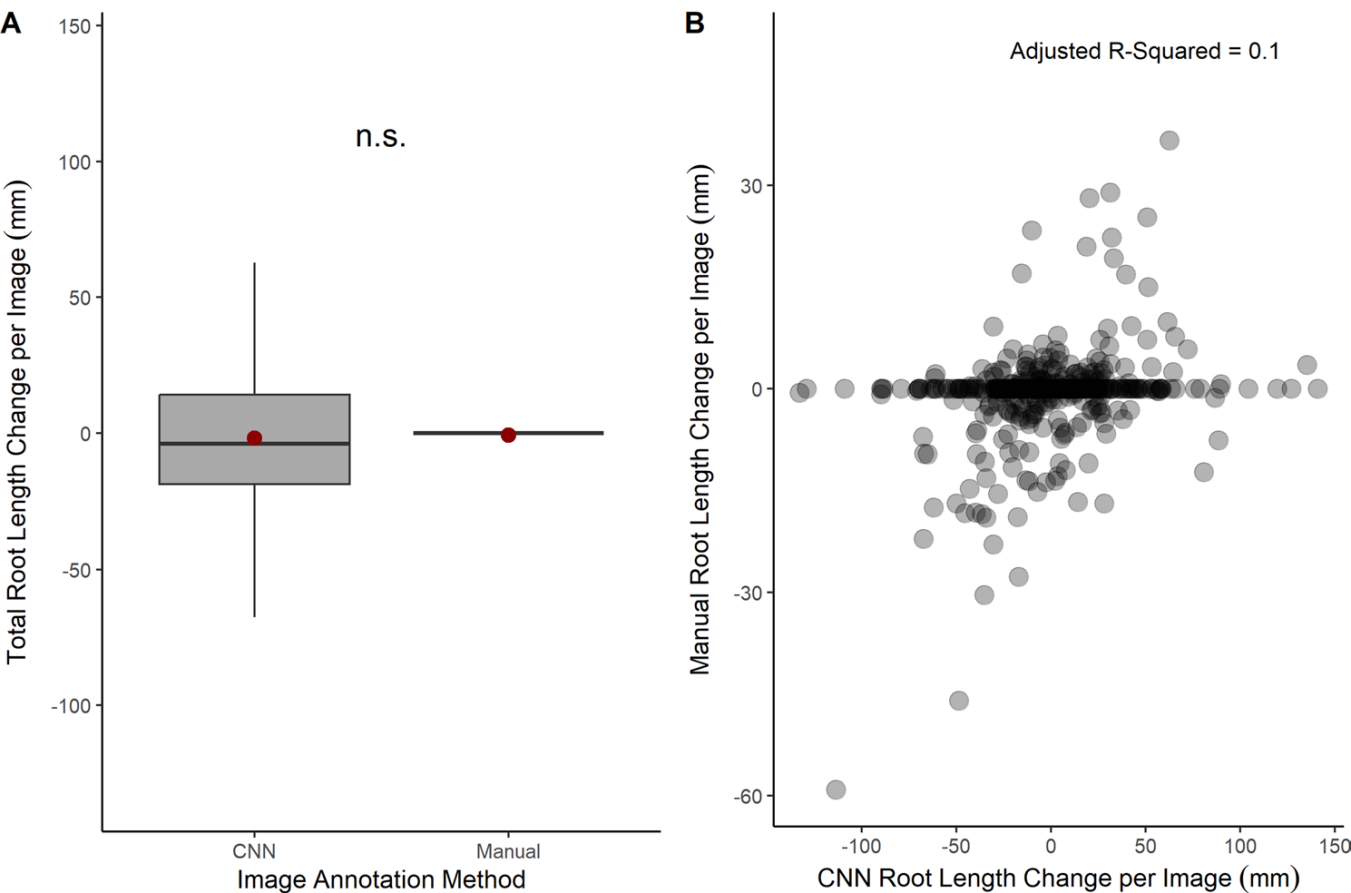
Root length change estimation across a 4-month period by an expert manual annotation and CNN. **A** Root length change comparison from an expert manual annotation of minirhizotron images (*n* = 529) using Rootfly and from a CNN model trained using RootPainter. **B** The relationship between total root length change per image (*n* = 529) from an expert consensus manual annotation and from a CNN model trained using RootPainter. Adjusted r-squared = 0.1

The CNN model suggested a greater decrease in total root length net change (mean − 1.8 mm and median − 3.8 mm) over the 4-month period than the human annotation net change (mean − 0.7 mm and median 0 mm) and a greater variation in net change per image in the CNN measurements (Fig. 7). The difference between the total root length net change output of the two image analysis methods (v = 63714, *p* = 0.08, Fig. 7) was substantial and close to statistically significant (Fig. 7).

## Discussion

### Variation in manual annotation between individuals

Less experienced annotators consistently identified more root length than experienced annotators (Fig. 3). This is consistent with Peters et al. [32], building on the suggestion that observer bias is present in manual root image annotation and inconsistencies between and within studies could be driven largely by variability in individual knowledge, training, and experience. Root length annotation by the same annotator reduced significantly by about 8-fold after 3 months of practice but then plateaued, remaining more or less the same after 1 year. This highlights the need for the annotator to be fully trained before commencing annotation to ensure consistency across a long-term dataset, but encouragingly shows that 3 months working as a part time research assistant, in this case, is enough time whereafter further experience does make a statistical difference to skill in root length annotation.

Manual segmentation is also subjective regardless of experience level, as variation in root length outputs were also present between individuals within the same experience categories (Fig. 4). The variation between annotators was ~ 2.5 times larger in the Novice group but still present in the experienced group, which is consistent with previous research [32] and displays potential for variability between annotations even when exclusively considering experienced image annotators. The high degree of variability between and within experience groups strongly suggests that this image-analysis data challenge is not suitable for crowdsourcing [48, 49].

Manual image analysis in this section of the study was carried out on images at a single time point. Minirhizotron image analysis normally consists of analysing images taken at the same point over a time-series [18, 20, 21]. Growing roots across a time-series would be easier to detect which would likely reduce the variation seen between manual annotations. It could also be that the high levels of complexity and heterogeneity displayed in this image dataset are a driver for this variation in annotation between experienced individuals [32]. Additionally, a lack of experience in a specific ecosystem could result in variation between even the most experienced minirhizotron image annotators i.e. individual previous minirhizotron image analysis experience likely focussed on one ecosystem that was different to the temperate forest ecosystem used in this study. Furthermore, it could be said that root ecologists often have a wider knowledge of what to expect in the system prior to analysing minirhizotron images i.e. by analysing site specific soil core samples. Therefore, a lack of previous analysis experience in a temperate forest ecosystem and limited site knowledge likely contributed, alongside the heterogeneity seen in the image dataset, to the discrepancy seen between individuals within this study.

It is evident that there needs to be more consistency in manual image annotation between annotators as variation persistently occurs between individuals, regardless of experience level. A universally applied public protocol or methods paper that focuses on the process of manual minirhizotron annotation across a wide range of ecosystems is therefore recommended, to enhance data quality and reduce bias between and within studies.

### Variation between manual annotation and CNN performance

The CNN model overestimated mean and median root length in comparison to the expert manual annotation (Fig. 5). Differences in individual image annotation show mainly large positive, but also negative, deviations in the CNN annotation (Fig. 5). The CNN model cannot, therefore, be regarded as wholly successful in producing accurate root length data at a single point in time in this forest context. It is important to note that this study compared the CNN output with an expert manual consensus as a measure of accuracy. The CNN is therefore being validated against human best practice which is considered to be the best representation of ‘ground truth’.

The relative weakness of the CNN to produce accurate root length data at a single point in time contrasts previous studies which have presented successful automation of root annotation using CNNs both trained [26, 34, 38] and not trained using RootPainter [29, 32, 35–37]. However, most studies where CNN’s have been applied to root image analysis focussed on single crop species in agricultural soil in fields [26] or pot/laboratory experiments [29, 35–37]. This likely resulted in image datasets featuring more homogenous mineral soils and rooting profiles and a rarity in the more complex, heterogenous images that are present in the forest image datasets used in this study. The impossibility to ensure perfect contact between minirhizotron tube and soil has the potential negatively affect image quality through precipitation and streaking/smearing on the outside of the tube. This may cause of over- or under-estimation by the CNN model in any type of ecosystem using minirhizotrons for data collection, not exclusively complex ecosystems like that used in this study.

The RootPainter trained CNN in Smith et al. [38] exhibited some over- and under-estimation of root length in more complex images featuring ‘bunching’ of roots, suggesting, not unexpectedly, that increased image complexity reduces model accuracy even in homogenous soil profiles. A good correlation between expert human annotation and output of a CNN trained in RootPainter has previously been reported in natural ecosystems (mediterranean tree-grass and temperate grassland) (*R*^*2*^ = 81% and 87%), but there remained consistent overestimation by the model [34]. This mirrors the overestimation seen in this study and reiterates that a CNN model trained using RootPainter does not perform as well when translated out of laboratory and agricultural sites and applied to more natural ecosystems with increased image complexity and heterogeneity.

A CNN model not trained using RootPainter contrasted the results of this study and showed high capability to correctly segment roots on minirhizotron images taken in multiple species, heterogeneous wetland substrate [32]. However, the specific neural network architecture used in RootPainter is a lower parameter U-Net model designed for fast convergence in an interactive training scenario [38]. Prior work has indicated that larger models with more parameters can reduce false positive errors in root segmentation in comparison to the network used in RootPainter [50], but this may result in a trade-off between a more accurate model and the accessibility of RootPainter to the average root scientist with limited machine learning experience.

Overestimation by the RootPainter CNN may also be related to how ambiguity is handled with the two different types of annotation in the RootPainter training process (dense vs corrective) [38]. When the user is annotating ‘manually’ i.e densely annotating the entire image, if they are unsure whether a structure is a root they may be conservative and not annotate it as a root when it is a root i.e. incorrect annotation of root absence. Corrective annotation on the other hand is to be assigned when the annotator sees a clear error [38]. Therefore, when the annotator is uncertain, it’s likely that many regions identified as root by the CNN are not corrected by the annotator to soil when they are in fact soil i.e. incorrect annotation of root presence. The difference in the way the decision is made between the two annotation styles represents the difference between an opt-in vs. opt-out approach to root annotation. This may provide some explanation for the model over-estimating roots in comparison to the manual annotation in this study. Additionally, extreme class imbalance bias in the dataset i.e. the majority of pixels in the images representing soil, means that there is more opportunity for false positives in the data than false negatives [50]. This again may contribute to the CNN model over rather than under-segmenting root length in this dataset.

A potential solution that may mitigate false positive errors whilst ensuring fast interactive training could be to use RootPainter alongside, rather than as a replacement for, manual annotation. This could involve manually removing all the images that do not contain roots as a pre-processing step to address some of the issues associated with class imbalance and over-estimation. Another solution could also be to finetune larger pre-trained models, using approaches similar to Chen et al. [39]. enabling both faster convergence and the benefits of larger network architectures in segmenting complex images with reduced false positives.

### Variation between manual annotation and CNN performance over a time series

There was consistent over estimation of root length in each individual month of the four-month time series (Fig. 6). The difference between the total root length net change output calculated over the 4-month period of the manual and CNN image analysis methods was also substantial and close to statistically significant, with the CNN reporting a greater decrease in total root length net change. However, the difference between the two methods’ outputs was much less for root length net change data (*p* = 0.08) (Fig. 7) than root length data at a single point in time (p = < 0.0005) (Fig. 5). This is likely because, whilst root length on an image may be over- or under-estimated at a single point in time, if the over- or under-estimation remains consistent across a period, there is potential for meaningful net root length change data to be a viable measure [34].

Although understanding net root length change is useful for root ecologists, no information can be acquired on the dynamics driving this change with current CNN models i.e. the respective contributions of root growth and mortality. To achieve the ability to acquire data on root growth, mortality and turnover rates, and subsequently gage a greater understanding of root dynamics, from a RootPainter trained CNN model, the potential to track single roots, as seen on manual annotation software such as Rootfly, would be highly beneficial [26].

### Applications and implications

The number of images used in training the model in this study was 766 more (*n* = 966 vs. *n* = 200), and time spent annotating was ~ 12 CPU hours more (n = ~ 15 vs. n = < 2), than in a previous example of successful model training using RootPainter [38], likely required due to the heterogeneous nature of the forest image dataset. However, the CPU-hours required to annotate root images using a CNN model trained using RootPainter is substantially less than manual annotation [26, 32, 38]. 15 CPU-hours were required to train this CNN model, whereas the manual annotation of the same 4 months of data took ~ 80 h. It is important to note that once the CNN is trained the time investment remains the same, whilst the time investment required for manual annotation increases with every new image taken i.e. 12 months of images in the same dataset would require the same 15 h of active training on RootPainter but an increase to 240 h of manual image analysis. If the trade-off between throughput and accuracy [26] is acceptable, AI approaches such as CNNs are attractive; in this study the accuracy of the CNN fell below our acceptance threshold, which was based on our experience of the data requirements in this ecological setting.

This study did not seek to make comparisons between different ecosystems, different minirhizotron cameras or different CNN software. If not bounded by practical limitations, these comparisons, alongside a larger cohort of manual annotators, would strengthen the research conclusions.

## Conclusions

The results of this study show that, in manual annotation, the same annotator who is fully trained on analysing minirhizotron images before analysis begins and has a full understanding of the system in question, should be used throughout studies of highly heterogeneous and class-imbalanced images (i.e., few targets amongst much noisy background) such as forest root image sets from minirhizotrons. As it is not always viable to use the same individuals within and between the studies, the value of highly yet consistently trained individuals is clear. This reiterates the need for a universally applied public protocol on minirhizotron image analysis to reduce the impact of bias within and between studies. Crowdsourcing, or naive applications of AI are very unlikely to provide results with sufficient accuracy and precision.

The literature suggests that, when a CNN model can be trained successfully on a set of images from the specific site in question, it is the obvious choice over manual annotation for minirhizotron root image analysis. They have been reported to greatly reduce the time investment required and have the potential to overcome the effects of observer bias on data outputs, especially in regard to long term datasets. However, this study finds that, despite ~ 160 person-hours and ~ 15 CPU hours of testing and refining, this CNN model cannot yet be relied on to produce root length data of sufficient accuracy and precision for ecological applications of the kind tested here. The model may still produce meaningful measures of relative change, but the trade-off between accessibility to researchers without a machine-learning background and the accuracy and precision of the results remains too strong for the analysis of images taken in natural ecosystems of a high level of complexity and heterogeneity. AI-enhanced manual segmentation may be the best option in the forest setting given the current state of the AI technology but would require development of a robust analytics pipeline linking the two processes.

The development and application of CNNs have greatly improved the accuracy and feasibility of minirhizotron image analysis for agricultural and lab-based environments thus far, but a continuing evaluation and development of accessible CNNs for natural ecosystems is required for the understanding and restoration of ecological systems in the face of global change.

## Data Availability

Upon publication, the datasets generated and analysed during this study will be available in the Zenodo repository 10.5281/zenodo.12804786. For the sake of the review, the datasets have been added to the submission as related files.
